# EXPECTATIONS FOR CHANGE IN SEXUAL POSSIBLE SELVES: IMPLICATIONS FOR SEXUAL WELL-BEING WITH AGE

**DOI:** 10.1093/geroni/igae098.2864

**Published:** 2024-12-31

**Authors:** Allyson Graf, Sydney Ellis

**Affiliations:** Northern Kentucky University, Highland Heights, Kentucky, United States; Northern Kentucky University, Highland Heights, Kentucky, United States

## Abstract

The concept of sexual possible selves is well-established in the emerging adulthood literature with connections to sexual behavior and wellbeing. Research on sexual possible selves beyond this age period is limited. In an online sample of adults (N = 612; Mage = 47.31, SD = 13.51, 30-85 years), the current study aimed to identify expectations for long-term change in sexual possible selves and understand related factors. Participants reported on expectations for change (no change, change a little, change a lot) and were asked to elaborate. Additional data were collected on demographics, self-rated health, and sexual interest as possible related variables. Although 40.4% of the sample indicated that they expected no change, 78.9% of participants described a long-term expectation for their sexual possible selves. Thirteen themes emerged; the most common were aging (23.4%), relationship with a current partner (22.6%), and partner-related issues including no partner (19.5%). A series of logistic regressions were run to identify factors related to each theme. Demographics including age and partner status largely predicted themes. A multinomial regression was run to explore factors associated with expectations of staying the same (40.4%), changing a little (47.0%), and changing a lot (12.6%). Although demographics again were significant, additional variables contributed and the pattern of variables differentiating no change from change a little and change a lot differed. The results are important for extending sexual possible selves research across adulthood and understanding the diversity of expectations that may influence sexual interactions and wellbeing with age.

